# The Role of Alcohol Consumption in Regulating Circulating Levels of Adiponectin: A Prospective Cohort Study

**DOI:** 10.1210/jc.2015-1845

**Published:** 2015-05-22

**Authors:** Steven Bell, Annie Britton

**Affiliations:** Research Department of Epidemiology and Public Health, University College London, London WC1E 6BT, United Kingdom

## Abstract

**Context:**

The role of alcohol intake in influencing longitudinal trajectories of adiponectin is unclear.

**Objective:**

The objective of the study was to examine the association between alcohol intake and changes in the circulating levels of adiponectin over repeat measures.

**Design, Setting, and Participants:**

A prospective cohort study of 2855 men and women (74% men with a mean age of 50 y at baseline) drawn from the Whitehall II study. Data from study phases 3 (1991–1993), 5 (1997–1999), and 7 (2002–2004) were used.

**Main Outcome Measure:**

Adiponectin serum concentrations (nanograms per milliliter) were measured, and alcohol intake was defined in terms of number of UK units (1 U = 8 g ethanol) consumed in the previous 7 days on three occasions. Cross-sectional associations between alcohol and adiponectin levels were calculated using linear regression. A bivariate dual-change score model was used to estimate the effect of alcohol intake on upcoming change in adiponectin. Models were adjusted for age, sex, ethnicity, and smoking status.

**Results:**

Alcohol consumption was cross-sectionally associated with (log transformed) adiponectin levels (β ranging from .001 to .004, depending on phase and level of adjustment) but was not associated with changes in adiponectin levels over time [γ = −0.002 (SE 0.002), *P* = 0.246].

**Conclusion:**

Alcohol intake is not associated with changes in circulating adiponectin levels in this cohort. This finding provides evidence that adiponectin levels are unlikely to mediate the relationship between moderate alcohol consumption and reduced risk of type 2 diabetes. It is important to consider dynamic longitudinal relationships rather than cross-sectional associations.

Moderate alcohol intake is associated with a lower risk of developing type 2 diabetes (1), and part of this effect is thought to be mediated via its role in increasing adiponectin levels (2–6). Higher levels of circulating adiponectin are alleged to be associated with a lower risk of type 2 diabetes (7) and prediabetes (8) in addition to cardiovascular disease (9), various forms of cancer (10), and major depression (11).

However, most studies linking alcohol intake to adiponectin rely on only one measure of alcohol consumption at baseline and adiponectin level ascertained either cross-sectionally or at a single follow-up occasion. It is important to consider the longitudinal development of both processes to determine how, if at all, the two are related. However, studies with repeat measures of alcohol consumption and adiponectin are scarce, so few studies have been able to examine the relationship simultaneously. One study found that changes in drinking over a 4-year period, specifically the uptake of modest drinking among initial nondrinkers and small increases in consumption among light drinkers, were associated with higher adiponectin levels (12). However, this study was reliant on a single measure of adiponectin at follow-up among only 697 men. Neither alcohol intake nor circulating levels of adiponectin are static processes (13, 14). That is, both change over time, and it is possible that accounting for the dynamic association between the two will shed additional light on the role of alcohol intake in regulating adiponectin concentrations. The purpose of this study was therefore to investigate how prospectively measured alcohol consumption is related to changes in adiponectin levels over repeat measures.

## Materials and Methods

### Study design and sample

Participants were drawn from the Whitehall II prospective cohort study (15). The study began in 1985–1988 (phase 1) and included 10 308 (6895 men) British civil servants aged 35–55 years. We present data at phases 3 (1991–1993), 5 (1997–1999), and 7 (2002–2004) from a diabetes case-cohort sample (14, 16) with measurements of adiponectin (n = 3477 with at least one valid measure). We excluded those with prevalent diabetes at baseline (n = 17). Furthermore we limited our sample to those who consumed alcohol at some point during follow-up to limit biases associated with lifelong nondrinking and sick-quitting prior to baseline influencing our estimates (exclusion of n = 110) (17, 18). Those with missing data on covariates were also excluded from the analytic sample (n = 530), resulting in a final sample size of 2855 individuals (note missing data counts for categories above are not mutually exclusive). Participants excluded from the analytic sample tended to be older, from lower socioeconomic groups, and of nonwhite ethnicity (there was no significant gender difference between excluded and included participants; data not presented).

The study was approved by the University College London Medical School Committee on the Ethics of Human Research. Informed consent was obtained at baseline and renewed at each contact. Whitehall II data, protocols, and other metadata are available to bona fide researchers for research purposes. Please refer to the Whitehall II data sharing policy at http://www.ucl.ac.uk/whitehallII/data-sharing.

### Measurements

#### Alcohol intake

Participants were asked to report the number of alcoholic drinks they had consumed in the previous week, providing information separately for beer/cider (pints), wine (glasses), and spirits (measures). Drinks were converted into UK units of alcohol (1 U is equivalent to 8 g of ethanol) using a conservative estimate of one UK unit for each measure of spirits and glass of wine, and two UK units for each pint of beer. The sum of these converted measurements was used to define total weekly number of UK units consumed.

#### Adiponectin

Adiponectin serum concentrations (nanograms per milliliter) were measured using the Quantikine ELISA kit (R&D Systems). The same standard operating procedures were followed for blood collection, processing, and storage during all study phases. Venous fasting (≥5 h of fasting) blood samples were drawn before a standard 2-hour oral glucose tolerance test. Within an hour, samples were centrifuged on-site and serum immediately removed from the monovette tubes into microtubes stored at −80°C. All assays were performed in the same laboratory (German Diabetes Center), and to minimize imprecision, samples from different study phases of the same participant were measured using the same ELISA plate. The limit of detection was 3.9 ng/mL (all samples gave values above the limit of detection).

#### Other covariates

We regressed the intercept and slope terms for both alcohol intake and adiponectin on the following time-invariant covariates: age at baseline (centered on the sample mean), sex, ethnicity (white vs nonwhite), and socioeconomic position defined using employment grade (high, intermediate, or low). We entered smoking status (not current vs current) as a time-varying covariate influencing adiponectin levels at each time point (19). We chose not to adjust for variables that may lie on the causal pathway between alcohol intake and adiponectin levels to avoid overadjustment bias (20, 21); this includes body mass index (22) and fasting insulin (23). Due to our sample size and the complexity of our longitudinal model, we did not stratify by sex or ethnicity.

### Statistical analysis

The association between adiponectin and alcohol intake cross-sectionally at each study phase was calculated using linear regression. To examine the association between adiponectin concentrations and weekly alcohol intake over repeat measures, we used bivariate dual-change score (BDCS) modeling, which allows for growth/decline to be measured while simultaneously allowing for lagged effects from one process on the upcoming change in the other variable. A detailed explanation of the mathematical and statistical properties of BDCS models can be found elsewhere (24, 25).

Briefly, change in a variable (Δ) is considered as a function of three components: 1) a constant amount (α), which is the sum of change scores over time; 2) an amount proportional to the previous value of itself (β), in many ways representing self-feedback in the dynamic system; and 3) an amount proportional to the previous state of the alternative variable (γ). It is also important to note that although BDCS models are usually specified as linear models (ie, the association between alcohol intake and changes in adiponectin is linear), nonlinear trajectories can be accommodated/modeled because at each time point the autoproportional (β) and coupling (γ) parameters are multiplied by the scores from the previous measurement occasion, which alter over time. The result is that even in a model in which the coefficients are assumed to be static over time, the actual effects are compounded across occasions as a result of being multiplied by shifting values (25, 26).

**Figure 1.**
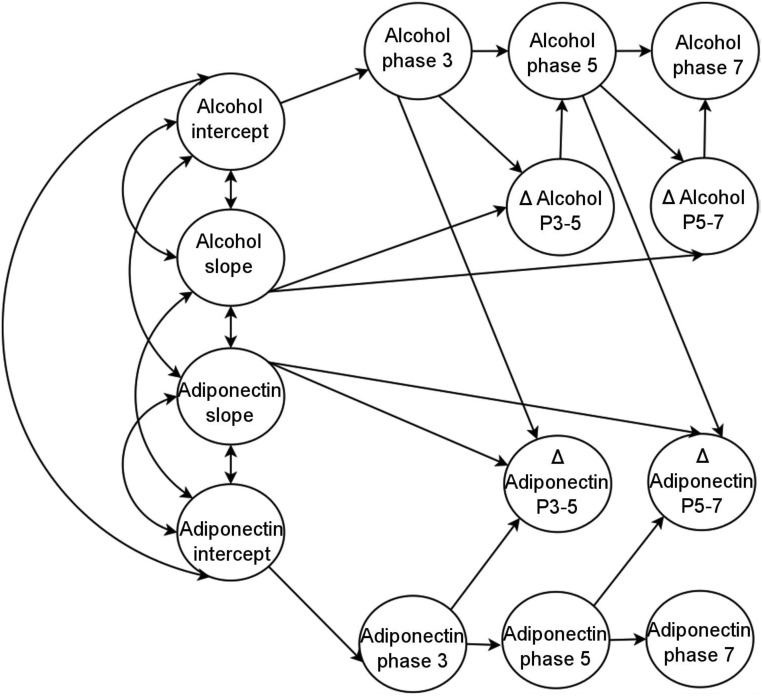
Simplified diagram of model specification. Single-headed arrows indicate regression coefficients, and double-headed arrows indicate covariance terms.

Both the intercepts (estimated values for log transformed adiponectin and weekly alcohol intake at the first study phase) and slopes (α terms) were fitted as random effects. Intercepts and slopes were correlated within single processes (for example, the adiponectin intercept with the adiponectin slope) and between processes (for example, the alcohol intercept with the adiponectin slope). See Figure 1 for a simplified graphical depiction of the model. As described above, intercepts and slopes were estimated conditional on baseline covariates, whereas smoking status was entered into the model as a time-varying covariate. Because adiponectin values were heavily positively skewed, we used natural log-transformed values for analysis.

Models were estimated in Mplus version 7.3 (27) using full information maximum likelihood with robust SEs. Model fit was examined using the Tucker-Lewis Index, the comparative fit index, and the root mean squared error of approximation. Cutoff values approaching 0.95 were used to determine a good fit for the Tucker-Lewis Index and the comparative fit index, whereas a threshold close to 0.06 was used for the root mean squared error of approximation (28). Statistical significance was inferred at a two-tailed *P* < .05.

## Results

### Descriptive statistics

Presented in Table 1 are the basic demographic statistics of the analytic sample. The mean age of participants at baseline was approximately 50 years (range 40–63 y). Almost three-quarters of the sample were men and most of them white (∼93%) and of high to intermediate socioeconomic position. Descriptive statistics concerning the primary variables of interest, alcohol intake and adiponectin, are presented in Table 2 alongside summaries of the proportion of current smokers, which also changed over time in the models estimated. Most the sample were nonsmokers and the prevalence of current smoking declined over time. Mean alcohol intake at baseline was almost 11 UK units per week, peaking at 14 U during follow-up before declining after this [consistent with previous work (13)]. Mean adiponectin levels declined throughout follow-up (geometric means of 9.06, 9.05, and 9.03 at study phases 3, 5, and 7, respectively).

**Table 1.** Basic Demographic Information of the Sample

Variable	n	Percentage or Mean (SD)
Age	2855	49.8 (6.0)
Sex		
Men	2107	73.8
Women	748	26.2
Ethnicity		
White	2650	92.8
Nonwhite	205	7.2
Socioeconomic position		
High	1193	41.8
Intermediate	1331	46.6
Low	331	11.6

**Table 2.** Descriptive Information for Variables Changing Over Time

	Phase 3		Phase 5		Phase 7		Within-Subject Standard Deviation
	n	Percentage or Mean (SD)	n	Percentage or Mean (SD)	n	Percentage or Mean (SD)	
UK units	2854	10.7 (12.6)	2795	14.3 (15.5)	2828	12.5 (13.1)	5.0
Adiponectin^a^	2855	9.1 (1.1)	2855	9.1 (1.1)	2855	9.0 (1.06)	0.2
Smoking status							
Not current	2855	88.1	2590	90.7	2636	92.3	—
Current	340	11.9	265	9.3	219	7.7	—

Dashes indicate not calculated.

a Geometric mean.

### Regression estimates

Fit indices for all estimated models fell within the acceptable ranges reported above (data not shown).

Presented in Table 3 are regression coefficients and SEs from a series of linear regression models of the cross-sectional association between alcohol intake and log-transformed adiponectin levels. In both age and sex as well as fully adjusted models, higher alcohol intake was associated with higher levels of circulating adiponectin [β ranging from 0.001 to 0.004, depending on the phase and level of adjustment; only the phase 3 fully adjusted did not meet the threshold for statistical significance (*P* = .12)].

**Table 3.** Regression Coefficients (SE) for the Association Between Alcohol Intake and Adiponectin Levels Cross-Sectionally at each Study Phase

Differences in Log (Adiponectin)	Age and Sex Adjusted	Fully Adjusted
Phase 3 alcohol (n = 2847)	0.002 (0.001)^a^	0.001 (0.001)
Phase 5 alcohol (n = 2700)	0.002 (0.001)^b^	0.001 (0.001)^b^
Phase 7 alcohol (n = 2644)	0.004 (0.001)^c^	0.003 (0.001)^c^

Fully adjusted means age, sex, ethnicity, socioeconomic position, and smoking status.

a *P* < .05

b *P* < .01.

c *P* < .001.

Table 4 contains regression coefficients and SEs for two bivariate dual-change score models, one with adjustment for age and sex only and another with adjustment for ethnicity, socioeconomic position, and changes in smoking status. Alcohol intake was significantly associated with upcoming changes in itself in both models [β = −1.642 (SE 0.121) in age and sex adjusted, and β = −1.647 (SE 0.123) in the fully adjusted model]. Adjustment for additional confounding factors attenuated the estimated lagged effect of adiponectin toward the null (β = 0.245 in the age and sex adjusted model compared with β = −0.047); however, in both cases the association was not statistically significant.

**Table 4.** Regression Coefficients (SE) for Bivariate Dual-Change Score Model of Alcohol Intake Affecting Upcoming Change in Adiponectin Levels

Alcohol → δ Adiponectin	Age and Sex Adjusted		Fully Adjusted	
	Alcohol	Adiponectin	Alcohol	Adiponectin
Fixed effects				
Intercept	12.757 (0.375)^a^	8.950 (0.010)^a^	13.839 (0.425)^a^	8.972 (0.012)
Slope (α)	25.028 (1.914)^a^	−2.186 (2.318)	26.953 (2.059)^a^	0.444 (2.034)
Autoproportional (β)	−1.642 (0.121)^a^	0.245 (0.258)	−1.647 (0.123)^a^	−0.047 (0.226)
Coupling (γ)	−0.001 (0.002)	—	−0.002 (0.002)	—
Random effects				
Intercept/slope covariance	184.031^a^	−0.044	180.918^a^	0.011
Intercept covariance	0.242^b^		0.143	
Slope covariance	0.318		0.595	
Alcohol intercept, adiponectin slope covariance	0.160		0.287	
Adiponectin intercept, alcohol slope covariance	0.533^c^		0.378^b^	

Fully adjusted means age, sex, ethnicity, socioeconomic position, and smoking status. The total number was 2855. Dashes indicate not calculated.

a *P* < .001.

b *P* < .05

c *P* < .01.

The effect of alcohol intake on upcoming change in adiponectin was nonsignificant in both models [γ = −0.001 (SE 0.002) in age and sex adjusted, and γ = −0.002 (SE 0.002) in the fully adjusted model].

### Conclusions

#### Summary of findings

Higher alcohol intake was associated with increased levels of adiponectin when measured cross-sectionally at all occasions; however, we found no evidence that alcohol consumption is associated with changes in circulating levels of adiponectin over a 10-year period in a well-documented middle-age cohort of mostly white men and women.

#### Comparison with previous work

Our cross-sectional findings are broadly in agreement with existing studies on the topic of alcohol intake and adiponectin, including interventional studies (6); however, our longitudinal findings are not in line with other observational studies (12). The existing longitudinal studies have typically examined the impact of a change in alcohol consumption between two measurement occasions on adiponectin levels at a single point in time. In contrast, our primary focus was on predicting the impact of alcohol consumption on changes in adiponectin levels over time. As such, our findings are not directly comparable. Whereas experimental studies have generally shown an association between alcohol intake and higher adiponectin levels, it is important to note that these effects are limited to the short term and there is substantial heterogeneity between them (6). It may therefore be that alcohol consumption is predictive of adiponectin levels acutely but not long term, and our findings are broadly supportive of this.

Adiponectin was one of several plausible biomarkers recently put forward as having compelling evidence in favor of it being a mediator in the relationship between moderate alcohol intake and reduced risk of coronary heart disease and related conditions (11). Our findings cast doubt on this assertion and add to the suspicion that a substantial proportion of the alleged protective effects of moderate alcohol intake can be explained by misclassification bias, residual confounding, and failing to account for longitudinal dynamics between alcohol consumption and health over time (13, 22, 29).

The role of adiponectin as an intermediate in the association between moderate alcohol intake and reduced risk of developing type 2 diabetes is further weakened when considering evidence from a large-scale Mendelian randomization study that demonstrated that adiponectin is unlikely to be causally associated with type 2 diabetes (30) [a recent meta-analysis also revealed that adiponectin levels are not predictive of coronary heart disease either (31)].

### Strengths and limitations

Our study is the largest investigation into the role of alcohol consumption on changes in adiponectin that we are aware of, with a sample size of 2855 men and women compared with 697 men (12). Unlike other studies, we were also able to use repeat measures of both alcohol intake and adiponectin. This is important because others have shown that accounting for variation in drinking over time is important when predicting health outcomes (13, 32).

Our study also has a number of limitations. For example, the Whitehall II study is not representative of the general population, so there may be concerns regarding the generalizability of our findings to the general population. However, it has been shown that etiological associations observed in Whitehall II are comparable with those observed in representative samples (33).

We also concentrated on total adiponectin level, but others have noted that multimetric forms of adiponectin exist (eg, high molecular weight oligomers, trimers, and hexamers) and the association between adiponectin levels and subsequent harm might be dependent on these different forms (21). Unfortunately, we did not have information on this. However, this is a shared limitation with previous work looking at alcohol intake and adiponectin so should not impact comparisons made between our work and the existing evidence base.

We also did not take into account beverage type; however, previous work has shown that beverage preference is not associated with the development of type 2 diabetes (34), and others have noted that often beverage-specific effects are likely to be confounded by socioeconomic position (35–37).

Finally, we considered only total weekly alcohol intake. Although this does not affect comparisons between our work and existing studies that have used similar measures (12), it is nevertheless a limitation because others have shown that drinking pattern is an important determinant of harm. We were unable to account for variation due to pattern of alcohol use per occasion (ie, someone drinking 14 UK units per week may consume 2 UK units per day over the course of a week or alternatively reach their total intake by consuming 7 UK units on two occasions); furthermore, it has been demonstrated that even irregular bouts of heavy drinking among typically moderate drinkers is associated with an increased risk of ill health (38).

### Concluding remarks

We found that average weekly alcohol intake is associated with higher levels of adiponectin cross-sectionally but is not associated with changes in total circulating adiponectin levels over time. Future work should examine the role of the drinking pattern in the association between alcohol intake and adiponectin as well as different forms of adiponectin.
